# A Silicon Photonics Computational Lensless Active-Flat-Optics Imaging System

**DOI:** 10.1038/s41598-020-58027-1

**Published:** 2020-02-03

**Authors:** Alexander White, Parham Khial, Fariborz Salehi, Babak Hassibi, Ali Hajimiri

**Affiliations:** Department of Electrical Engineering, California Institute of Technology, Pasadena, CA USA

**Keywords:** Electrical and electronic engineering, Integrated optics, Computational science, Imaging and sensing

## Abstract

The need for lightweight, miniature imaging systems is becoming increasingly prevalent in light of the development of wearable electronics, IoT devices, and drones. Computational imaging enables new types of imaging systems that replace standard optical components like lenses with cleverly designed computational processes. Traditionally, many of these types of systems use conventional complementary metal oxide semiconductor (CMOS) or charge coupled device (CCD) sensors for data collection. While this allows for rapid development of large-scale systems, the lack of system-sensor co-design limits the compactness and performance. Here we propose integrated photonics as a candidate platform for the implementation of such co-integrated systems. Using grating couplers and co-designed computational processing in lieu of a lens, we demonstrate the use of silicon photonics as a viable platform for computational imaging with a prototype lensless imaging device. The proof-of-concept device has 20 sensors and a 45-degree field of view, and its optics and sensors are contained within a 2,000 *μm* × 200 *μm* × 20 *μm* volume.

## Introduction

Lightweight miniature imaging systems are becoming essential to the development of wearable electronics, IoT devices, and drones^1–3^. Computation can be used to estimate information that is not captured by the imager, as in phase-retrieval imaging^4^, three dimensional imaging^5^, and encoded/coded aperture imaging^6^. Cleverly designed computational processes can also be used in imaging systems to replace standard optical components like lenses^7^. Traditionally, many of these types of systems use conventional CMOS or CCD sensors for data collection^4,6,8^. While the use of these prepackaged sensors allows for rapid development of large-scale systems, it fails to realize the full potential of a co-designed system-sensor in terms of versatility, performance, size, and mass^5,9^.

In this paper, we present an imaging system based on a custom-designed array of unique diffraction gratings (acting as optical receivers) co-designed with an adaptive reconstruction algorithm and implemented on an integrated photonics platform. This approach enables imaging within a very small volume, without the need for the depth typically required for most imaging systems. Integrated photonics allows for the capturing, manipulation, and sensing of light within an active layer only a few microns thick on a chip using dielectric waveguides manufactured with standard CMOS-type processing^10^. Furthermore, the built-in directionality of the custom integrated photonics microgratings can serve as a means of enhancing the computational image-recovery performance. These diffraction gratings can be engineered to have sensitivity in nearly arbitrary patterns and to admit a large span of wavelengths^11,12^, and as we show, can respond to incoherent light as well as coherent light. Using micrograting couplers co-designed with computational processing, we demonstrate that silicon photonics is a viable platform for computational imaging using a prototype lensless imaging device.

## Results

### Sensor design and implementation

Images are representations of light field patterns where each point represents the intensity (and possibly other properties, such as color) of light impinging on the imager from a different bearing. When an image is displayed, these points replicate the original light, and the eye can process the image similarly to the original scene, using a lens to direct light from each angle to a different photoreceptor cell on the retina. Conventional cameras work in the same way, using a lens to redirect light impinging at different angles onto different pixels. However, this is not the only way to capture an image. As long as enough independent information is captured from a light field pattern, an image can be formed. For instance, this can be achieved, as in the retinal cells in the compound eye of an insect, with sensors/pixels individually sensitive to light impinging from different directions^13,14^, or even in a different domain, such as Fourier space^15^. While the data itself is not in the form of an image, various computation algorithms can be used to generate a recognizable image from the captured information^7^.

Here, we use an array of individually designed silicon-on-insulator (SOI) diffraction gratings to capture spacial intensity information from incoherent light with a wavelength centered at 1550 *nm*. By using an array of custom micrograting couplers, each with a different sensitivity profile vs. angle of incidence, we can capture enough independent information to linearly map our data into spatial pixels.

**Figure 1:**
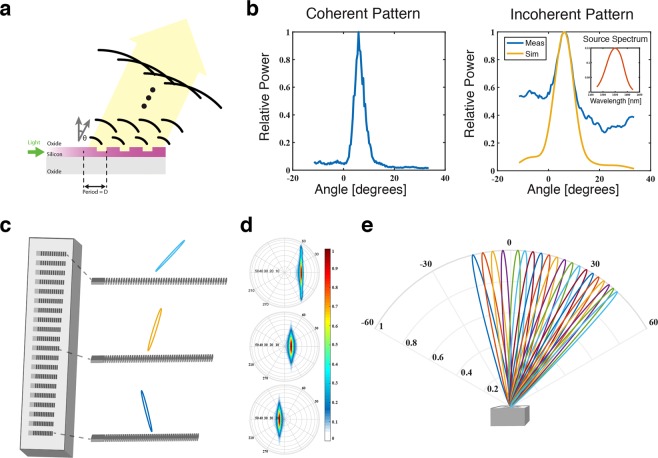
(**a**) Operation of a silicon photonics grating coupler. Light guided in a silicon waveguide clad by silicon dioxide is scattered by regularly spaced etched gratings. These scattered waves interfere coherently in a direction *θ* dependent on the period of gratings *D*. (**b**) The measured coherent and incoherent patterns of a grating coupler. The incoherent pattern is measured with the source spectrum shown. The simulated data is the theoretical pattern given the coherent pattern, the source pattern, and equation (5). (**c**) Visual representation of the fabricated device containing 20 uniquely spaced grating couplers. Three example couplers are shown with their simulated angular reception patterns. (**d**) Hemispherical projection of angular reception patterns of the corresponding grating couplers in c. (**e**) Simulated angular reception patterns of the full 20 grating couplers, in the direction of the gratings.

Micrograting couplers are versatile devices used to couple light from free space to guided modes in integrated photonics waveguides. Basic grating couplers consist of a periodic structure that diffracts incoming light such that it interferes coherently into the plane of the grating and a taper or other mode matching structure that guides the light into a waveguide mode, Fig. 1a. The periodicity and effective indexes of its internal components serve as design parameters that can be used to achieve the desired angular sensitivity profile of a coupler. For instance, in the case of uniformly spaced grating elements, the angle of maximum sensitivity is given by:

1 $${\theta }_{max}=si{n}^{-1}\left({n}_{eff}-\frac{k{\lambda }_{0}}{d}\right)$$

where *n*_*eff*_ is the effective index of the grating structure, *λ*_0_ is the free space wavelength, *d* is the distance between gratings, and *k* is any integer.

The one-dimensional radiation pattern of a uniform coupler in the direction parallel to its main axis can be approximated by assuming an exponential optical loss along the coupler in conjunction with the propagation phase shift:

2 $$h(\theta )\propto \sum _{k}{(1-\alpha )}^{k-1}{e}^{i\left(\frac{d}{{\lambda }_{e}ff}-\frac{d}{{\lambda }_{0}}sin(\theta )\right)}$$

where *α* is the loss per grating tooth and *λ*_*eff*_ is the effective wavelength inside the grating structure. Given such a pattern *h*(*θ*), the power received at time, *t*, due to an arbitrary farfield illumination *I*(*θ*, *t*) can be given as

3 $$P(t)=| {\int }_{\theta }h(\theta )I(\theta ,t)d\theta {| }^{2}$$

Under spatially incoherent illumination, *I*(*θ*) and ${I}^{* }(\theta ^{\prime} )$ are uncorrelated except when $\theta =\theta ^{\prime}$. If the illumination is also temporally incoherent, we can represent the total power as

4 $$P={\int }_{\theta }{\int }_{\omega }| h(\theta ,\omega ){| }^{2}| I(\theta ,\omega ){| }^{2}d\omega d\theta $$

with a single illumination source, we can separate the angular and frequency dependence of *I*, and define a modified sensitivity pattern ${h}^{^{\prime} }(\theta )$,

5 $$| h^{\prime} (\theta ){| }^{2}\equiv {\int }_{\omega }| h(\theta ,\omega ){| }^{2}S(\omega )d\omega $$

where *S*(*ω*) is the frequency spectrum of the scene illumination. Under narrow-band illumination, the pattern of a uniform coupler can be approximated as the convolution of the coherent pattern and the optical power spectrum, Fig. 1b.

For non-uniform couplers or extremely broadband illumination, the sensitivity pattern can be calculated by explicit simulation and weighted averaging of the patterns and the wavelength component of the optical power spectrum. As incoherent illumination leads to power addition and not electric field addition (as it would under coherent illumination), the power received by an arbitrary far-field distribution is given by the inner product of the sensitivity pattern and the far-field pattern. We can discretize this inner product to have the following approximation,

6 $$P\approx {\sum }_{\theta }| h^{\prime} (\theta ){| }^{2}| I(\theta ){| }^{2}\Delta \theta ,$$

where the number of discretization points determines the accuracy of our estimation. Consequently, we can rewrite (6) as the following,

7 $$P={{\bf{h}}}^{T}{\bf{x}},$$

where **h** is an *n* dimensional positive pattern vector (${\bf{h}}\in {{\mathbb{R}}}_{+}^{n}$), and **x** is the intensity vector, where *n* is given by the number of the distinct values of *θ* we have used in the approximation (6). If we combine the results from multiple sensors, we can get the following linear system of equations,

8 $${\bf{y}}={\bf{Hx}}+{\bf{z}},$$

where the *m*-dimensional positive vector **y** is the measured power, *m* is the number of sensors, the *m*-dimensional vector **z** is the unknown noise vector, and $${\bf{H}}=\left[\begin{array}{c}{{\bf{h}}}_{1}^{T}\\ {{\bf{h}}}_{2}^{T}\\ \vdots \\ {{\bf{h}}}_{m}^{T}\end{array}\right]$$ is the matrix whose rows correspond to the pattern of each sensor. As we construct **H** through the design of our grating couplers, we can reconstruct the image **x** by inverting the linear system.

To form an imager, we can modulate the grating pitch across a number of couplers, effectively generating a set which is sensitive to different illumination angles. In this way, the sensors mimic the effect of a lens on traditional pixels. As each grating coupler is designed to be most sensitive to a different angle, the approximated transfer matrix **H** (between discrete angles and sensor power) can be inverted effectively. Heuristically, overlapping sensor patterns allow for the data capture and localization of the full imaging area, and single angle reception allows for more independent measurements, improving the resolution or minimum resolvable angle. Thus it is favorable to strike a balance between overlapping and narrow sensor patterns.

As a proof of concept, we designed and fabricated a device with 20 of these sensors on a silicon photonics SOI platform, Fig. 1c. Each sensor consists of a uniform grating coupler and a germanium photodiode, sensitive to light with a wavelength around 1,550 *nm*. The grating couplers guide impinging light into 500 *nm* by 220 *nm* transverse-electric single mode silicon waveguides, which are then fed to the germanium photodiodes. The photodiodes convert the optical power received to an electric current, which can be amplified and measured. These sensors have a far-field coherent full-width half-maximum sensitivity of approximately 5 degrees in the grating direction and 30 degrees in the transverse direction, Fig. 1d, and an average peak efficiency of 21.5%. Due to reflections from the silicon substrate, the peak efficiency varies over the couplers, Supplemental Fig. 1. To minimize the effect of stray light and reflections, the photodiodes and area surrounding the grating couplers is covered by metal and an absorptive germanium layer. By sweeping the grating pitch, the maximum reception angle is swept from −10 to 30 degrees (to normal) in 2 degree increments, Fig. 1e.

**Figure 2:**
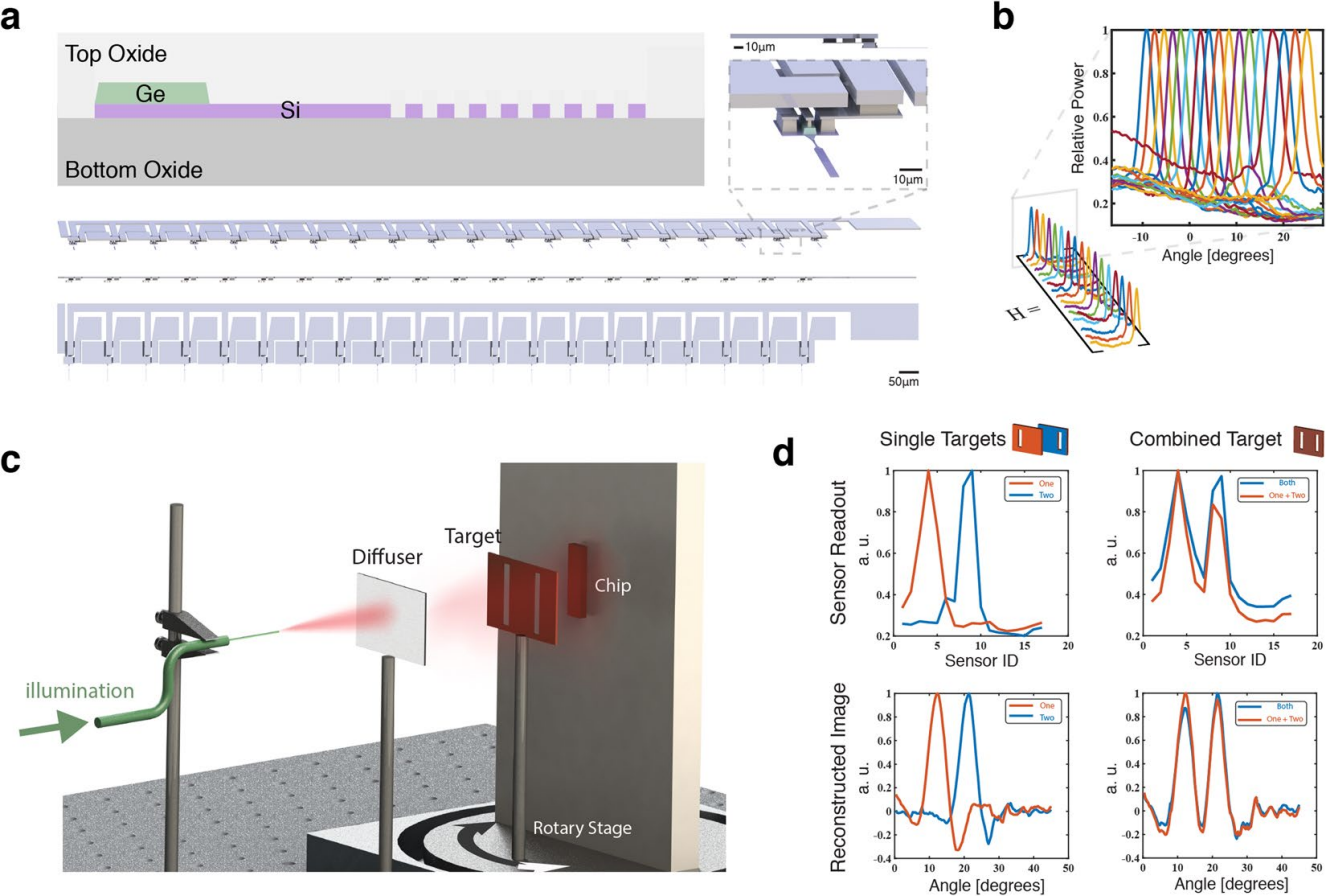
(**a**) Diagram of silicon on insulator platform with germanium photodiodes (top left). Scale isometric rendering of fabricated device from angle, sides, and top. Silicon shown in purple, germanium in green, and metal connections, pads, and vias in grey. (**b**) Measured angular reception patterns of the 17 functional sensors. Three sensors were not functioning due to photodiode connections. (**c**) Representation of test setup. The chip is illuminated through a cleaved optical fiber, diffuser, and target. The copper target blocks light from some the angles it is present, forming a negative of the barcode image seen by the chip. The target and chip are mounted to a rotary stage so that the light spread from the diffuser can be averaged to effectively provide an even illumination across all visible angles. (**d**) Data collected (top row) and reconstruction (bottom row) of three targets: two with slits in different positions (left column) and one with both slits (right column). (Graphics made by authors).

The fabricated device is shown in Fig. 2a. The reception patterns of 17 functional gratings were measured with a super luminescent diode (SLD) with a 50*nm* bandwidth, and are shown in Fig. 2b. To verify the functionality and linearity of the sensor, we imaged light impinging on a double slit and each of its constituents, Fig. 2c,d. By imaging two slits and their combination, we can test the approximation in Eq. 6, as the image formed by the combination should be the sum of the constituents. To do this, light from the SLD is first projected from a cleaved optical fiber onto a diffuser to perturb the spacial coherent of the beam. The diffuse light then travels through a copper complementary image (negative) of a target image and impinges on the chip. The target and chip are positioned on a rotary stage so that the illumination from the diffuser can be integrated over the entire angular sensitivity range (45 degrees). The final images can then be reconstructed by inverting the measurement in Fig. 1b, our new **H**, and multiplying by the data collected.

### Robust reconstruction algorithm

To capture images with this sensor, we must reconstruct the unknown intensity vector **x**, from the (noisy) linear system (8). This reconstruction problem faces two main challenges. First, because we need the discrete approximation in (6) to be accurate, the number of measurements *m* = 17 is much smaller than the underlying dimension *n* = 120. Second, our algorithm must be tuned properly to adjust for the impact of the noise in the linear system.

To address both of these challenges, we employ regularized least squares^16,17^, which provides us with the following estimate,

9 $$\widehat{{\bf{x}}}=arg\mathop{min}\limits_{{\bf{x}}}| | {\bf{y}}-{\bf{H}}{\bf{x}}| {| }^{2}+\lambda \,| | {\boldsymbol{\Gamma }}{\bf{x}}| {| }^{2}\,.$$

where **Γ** is an *n* × *n* matrix that is chosen based on the structure of the underlying dataset and *λ* is the regularization parameter that must be tuned properly. Note that the objective function in (9) consists of two terms. The first term in the objective function, the least-squares term, essentially captures the power of the noise vector **z**. The second term in the optimization, the regularization term, enforces a structure dictated by **Γ** on the unknown vector **x**.

For any set of parameters, we can represent the solution to the optimization (9) in the following closed-form,

10 $$\widehat{{\bf{x}}}=\left(\right.{{\bf{H}}}^{T}{\bf{H}}+\lambda {{\boldsymbol{\Gamma }}}^{T}{\boldsymbol{\Gamma }}{\left)\right.}^{-1}{{\bf{H}}}^{T}{\bf{y}}.$$

which leads to very computationally efficient solutions.

**Figure 3:**
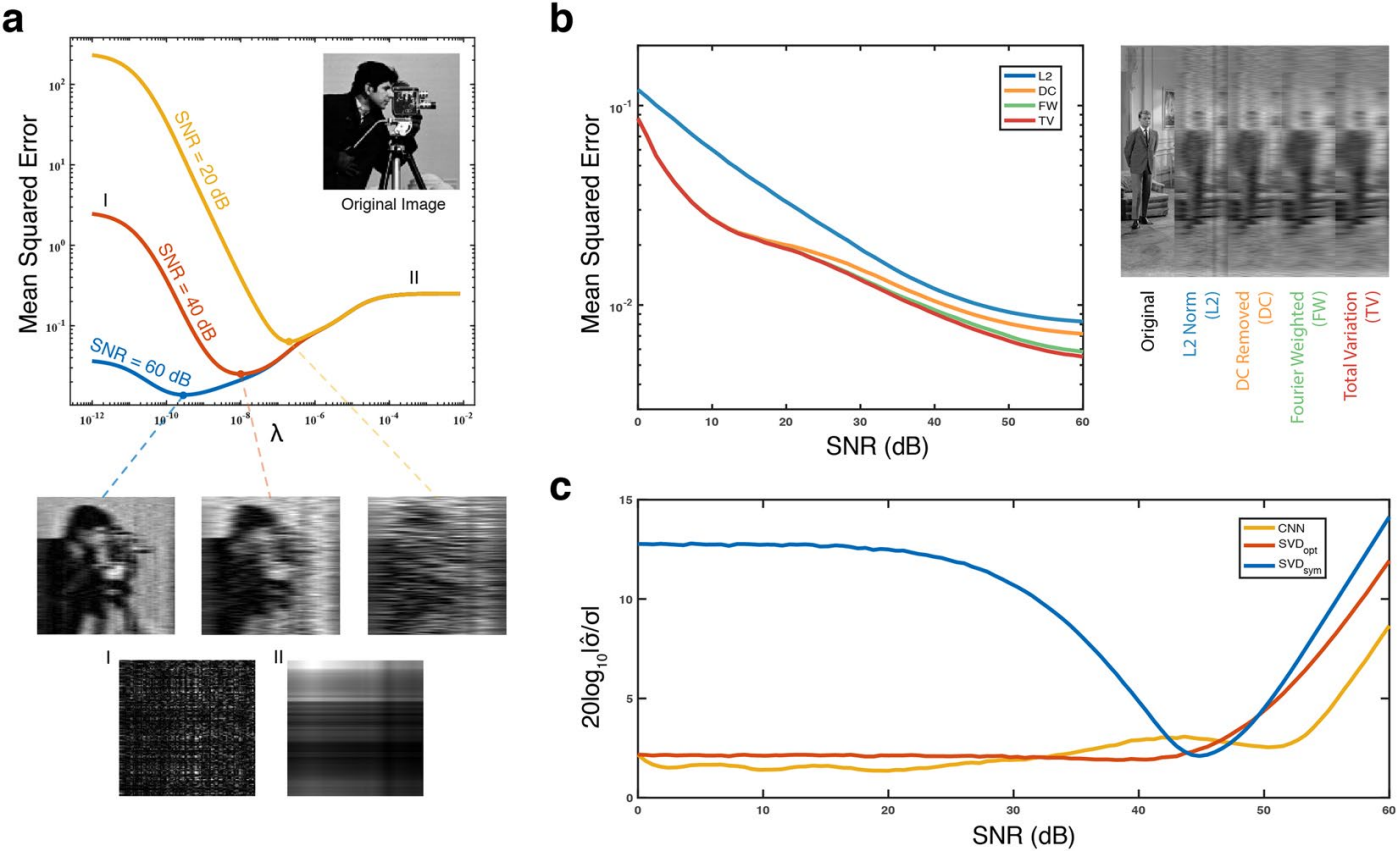
(**a**) Comparison of simulated reconstruction error across *λ* for data with different SNRs. Images shown for *λ*_*opt*_ of each SNR as well as in the low lambda and high lambda regimes. As this is a 1D imager, an image consists of a single slice of 1 × 120 pixels. The 2D images are representations of the concatenated 1D slices. (**b**) Comparison of reconstruction error with different **Γ** matrices at *λ*_*opt*_. (**c**) Comparison of SNR estimators: CNN, SVD with a heuristic filter (*SVD*_*sym*_), and SVD with an optimized filter (*SVD*_*opt*_). (Public domain images made available by the University of Granada Computer Vision Group).

To generate an optimal reconstruction, we must choose an optimal regularization strength *λ* and regularization matrix **Γ**. The choice of these parameters is critical, as can be seen in Fig. 3a.

The regularization matrix, **Γ**, should be chosen based on the structure of the underlying data^18^. As images do not follow any inherent rigid structure, there is no strictly optimal **Γ**, but we can use general properties of images to chose better matrices. Here, we utilize four matrices for **Γ** which have desirable properties for image reconstruction (detailed in Methods: Gamma Matrices): The identity or L2 norm (L2), DC removed (DC), Fourier Weighted (FW), and Total Variation (TV). Figure 3b shows a comparison of the reconstruction algorithms for the above choices of the matrix **Γ**.

After choosing a favorable regularization matrix, **Γ**, we must tune *λ* to its optimal value, *λ*_opt_. In the optimization program (9), it can be observed that the first term, ∥**y** − **Hx**∥^2^, measures the power of the noise vector, and the second term, i.e., ∥**Γx**∥^2^, measures the power of the signal. Hence, we expect the optimal value of the *λ* to be inversely proportional to the signal-to-noise ratio (SNR), i.e., ${\lambda }_{\mathrm{opt}}\propto \frac{1}{SNR}$. Extensive numerical simulation (see Supplemental Info) validates this fact. Thus to compute *λ*_opt_, we need to evaluate the SNR.

While the noise level could be calibrated, chip-to-chip variation, temperature fluctuation, varying levels of electrical interference, and numerous additional environmental factors can affect the noise present in a measurement. As optimal reconstruction is highly sensitive to SNR, to create a more robust system, we devise two algorithms to estimate the noise variance in a given measurement. Note that the noise in the system of equations (8) is caused by two different sources: 1) The measurement noise stemming from the device. Note that this noise can have impact of both **H** and the measurement vector **y**. 2) The approximation error caused by discretizing the model in (6). Note that in order to have a better approximation in the discrete model, one needs to increase the signal dimension *n*; however, this would have a negative impact on the reconstruction as $\frac{m}{n}$ decreases. For our analysis purposes, we assume that the additive noise, **z**, has independently and identically distributed Gaussian entries with mean zero and variance equal to *σ*^2^. In order to find an estimate for SNR we employ two different algorithms. Our first proposed algorithm relies on the assumption that the underlying signal **x** is smooth. This assumption is commonly used in imaging applications^18^. Consider the singular value decomposition of the measurement matrix **H** as follows,

11 $${\bf{H}}={\bf{U}}{\boldsymbol{\Sigma }}{{\bf{V}}}^{T},$$

where **U** is an *m* × *m* orthogonal matrix whose *i*^th^ column is represented by **u**_*i*_, **Σ** = diag(*s*_1_, *s*_2_, …, *s*_*m*_), and **V** is an *m* × *n* matrix with orthonormal columns, where **v**_*i*_ denotes its *i*^th^ column. We then consider the impact of the reconstruction algorithm on the noise vector. We have,

12 $${{\bf{z}}}^{\dagger }={{\bf{H}}}^{\dagger }{\bf{z}}={\sum }_{i=1}^{m}{s}_{i}^{-1}({{\bf{u}}}_{i}^{T}{\bf{z}}){{\bf{v}}}_{i}\,,$$

where **H**^†^ is the pseudo-inverse of **H**. If we filter out the low frequency components of this reconstruction (assuming that the underlying signal is smooth), we are left with an approximation of the reconstruction contribution of noise, and *σ*^2^ can be approximated as,

13 $${\widehat{\sigma }}^{2}=\frac{1}{m}{\sum }_{i=1}^{m}{({s}_{i}{{\bf{v}}}_{i}^{T}{{\bf{z}}}^{\dagger })}^{2}.$$

The second proposed algorithm is to train a convolutional neural network^19,20^. This approach is particularly useful when we have enough samples from the dataset. Unlike the first approach, here we do not need any assumption on the structure of the underlying signal. In our implementation, we trained a 4-layer network with two convolution layers and two fully-connected layers. The details of the training is deferred to Methods. Figure 3c compares the result of the above-mentioned algorithms in estimating the SNR. As expected, the CNN approach often provides a better estimate.

**Figure 4:**
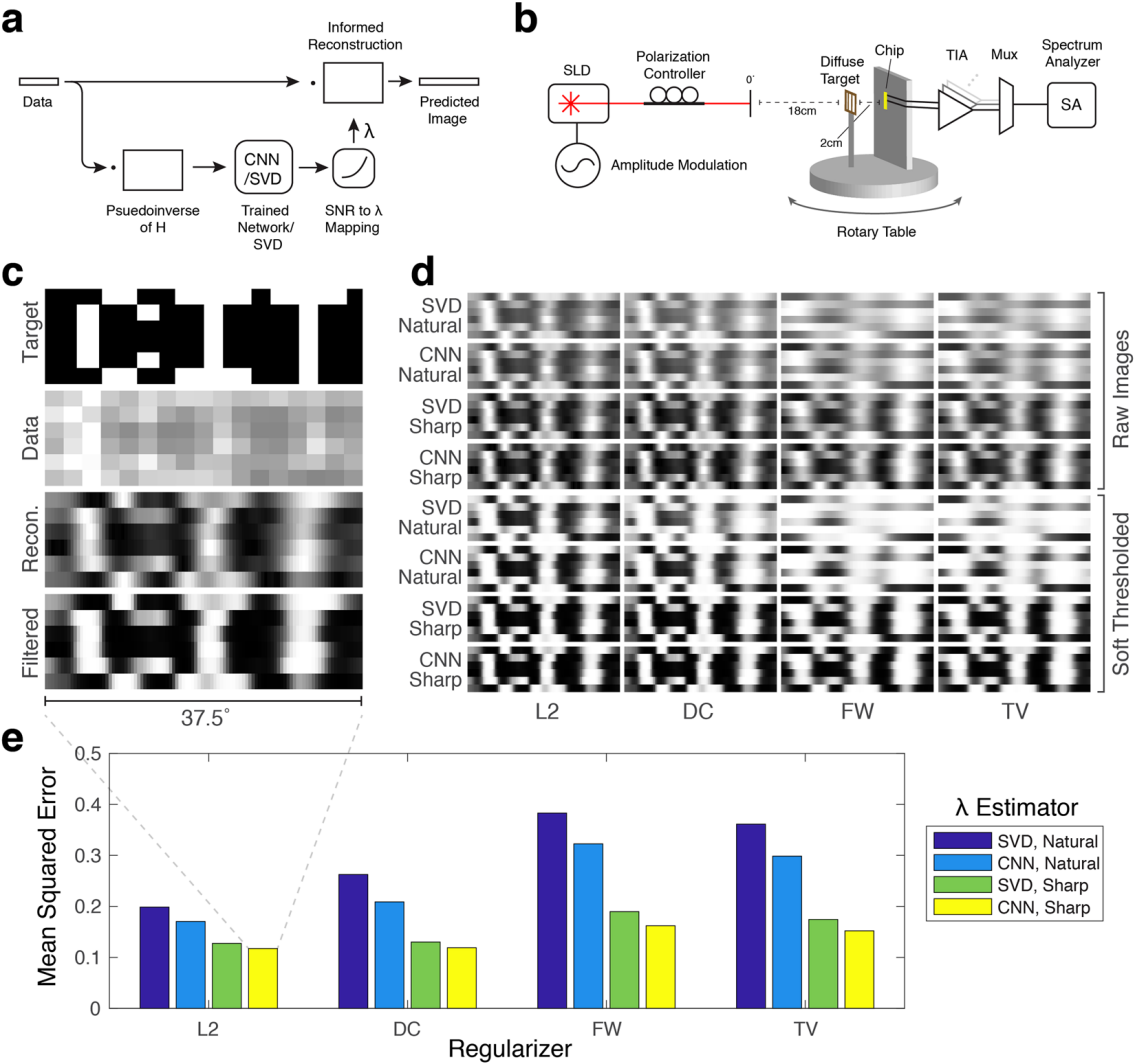
(**a**) Block diagram of reconstruction algorithm. (**b**) Measurement setup. (**c**) Reconstruction example with measured CIT barcode patterns. ’Target’ shows the barcodes used, displayed horizontally and concatenated vertically. ’Data’ shows the data collected from each of the 17 sensors. ’Recon.’ shows the image reconstructed using L2 regularization, CNN noise estimation, and an SNR-*λ* mapping for sharp images. ’Filtered’ shows the reconstruction after passing through a sigmoidal amplitude filter. (**d**) Reconstructed images using different **Γ** matrices, SNR estimators, and SNR to *λ*_*opt*_ mappings. ’Natural’ is using the SNR to *λ*_*opt*_ mapping optimized for natural images, and ’Sharp’ is using the SNR to *λ*_*opt*_ mapping optimized for random binary images. (**e**) Quantified reconstruction error for plots in d. Each bar represents the average of the 6 barcode patterns. (Graphics made by authors).

To form an image, the data collected from the sensor is fed through the SNR estimator, the SNR is mapped to *λ*_opt_, and the data is finally processed with the reconstruction (9), Fig. 4a.

### Measurement

To test the full system, we measure 6 barcode patterns, Fig. 4c (Target), that spell the letters C, I, T when concatenated. The collected sensor data is then multiplied by a reconstruction matrix **H** and fed through the trained CNN or SVD algorithm to estimate the noise variance. This noise variance is then used to map to an optimal lambda estimate through SNR. Finally, a new **H**_*opt*_ is calculated using this optimal lambda and the image is reconstructed, Fig. 4a. Figure 4c–e show the resulting images reconstructed using each of the four **Γ** matrices, SVD and CNN algorithms, and two SNR to *λ* mappings. We achieved a minimum mean squared error of 11.7%, equivalent to a peak SNR of 9.3 dB.

## Discussion

Here we develop an imaging technique that utilizes an engineered set of grating couplers in integrated photonics to capture images without the need for a lens or active beam-steering. We demonstrate this technique with a 1-dimensional imager that occupies only 2000 × 200 × 20 *μm*^3^ and can capture 20 data points a 45-degree field of view. This type of miniature image sensor could enable imaging in systems that require extremely light-weight and compact sensors, such as wearable electronics, drones, and health monitors. In addition, the demonstrated algorithms can be used in other computational imaging systems, and in many cases can easily coordinate with existing infrastructure using linear regularizers to improve robustness to noise.

Because it is implemented in an integrated photonics process, this type of sensor can be augmented by photonic processing techniques such as filtering and passive beam-forming^21–23^, and can be generalized to 2D and 3D imaging. By optimizing gratings for transverse as well as longitudinal angles, the same type of imaging can applied to a 2D field of view, and by using parallax or optimization of near field reception patterns, this type of imager could also capture 3D data. For high resolution in the extra dimensions, the imager system requires many more gratings, and thus larger sensors for a similar effective aperture. To get around this problem, it may be necessary to use grating couplers optimized for multiple gratings to increase the camera’s effective aperture. To reconstruct these 2D and 3D images, we can take the same algorithmic approach as an the 1D case by adding the extra dimensions in the transfer matrix H (replacing it with a tensor or flattened tensor). While the reconstruction will be more computationally intensive, the longer computational time can be shortened using pre-calculated matrix inverses. In addition, photonics information processing techniques could be used in as a prepossessing step or in conjunction with the reconstruction algorithm^24,25^.

## Methods

### Sensor characterization

The angular sensitivity of each grating coupler-photodiode pair was measured by recording the photodiode output current in each of 120 angles as set by a rotating table the chip was mounted to. The light source (laser or SLD) was projected from a cleaved fiber approximately 20*cm* from the chip surface. To improve SNR, the laser/SLD was modulated with a continuous wave and the amplified output current was read by a spectrum analyzer.

### Imaging targets

Targets were imaged as shown in Fig. 4b. Amplified and polarized SLD light was projected onto a ground glass and copper diffusive target with an optical power density of 0.51 *mW*∕*cm*^2^, illuminating the chip. The polarization of the projected light was aligned with the grating couplers to allow for maximum reception efficiency. Output power was measured for each of the 17 functional grating coupler-photodiode pairs in each of 40 angles spanning 50 degrees. These measurements were then averaged, effectively modeling a uniform illumination over the 45 degree field of view.

### Simulation data

To test the reconstruction algorithms, 18432 training and 6144 test images were used, formed by slicing 48 512 × 512 pixel images (graciously collected and made public by the Computer Vision Group at the University of Granada) into 1 × 120 pixel 1D images. To simulate data from the sensor, these images were multiplied by the measured **H** matrix and i.i.d. Gaussian noise was added to achieve the desired SNR.

### Gamma matrices

Here, we utilize the following matrices for **Γ**:

1. **Γ** = **I**_*n*_ : This correspond to least-squares with *ℓ*_2_ regularization. It has been observed that this regularization is useful as for stabilizing the solution of the least-squares.
2. **Γ** = **F**^*T*^**DF**: Here **F** is the *n* × *n* discrete Fourier transform and **D** = diag(*d*_1_, *d*_2_, …, *d*_*n*_) is a diagonal matrix. This choice of regularization matrix is particularly useful when we have some prior information about the frequency structure of the underlying signal. In our experiments we have exploited the following two choices of the matrix **D**:
  • $${d}_{i}=\left\{\begin{array}{l}0\,,\,\,\,\mathrm{for}\,i=1\,\\ 1\,,\,\,\,\mathrm{for}\,i\ne 1\,\end{array}\right.$$: This choice of the matrix **D** simply removes the DC-term of the signal in the regularization.
  • $${d}_{i}=\left\{\begin{array}{l}0\,,\,\,\,\mathrm{for}\,i=1\,\\ {(i+60/59)}^{2}\,,\,\,\,\mathrm{for}\,1 < i\le 60\,\\ {((121-i)+60/59)}^{2}\,,\,\,\,\mathrm{for}\,60 < i\le 120\,\end{array}\right.$$: This choice of the matrix **D** imposes different weight on different frequency components of the underlying signal such that the high-frequency components (indices close to $\frac{n}{2}$) have higher weights.
3. **Γ** = **Γ**_*TV*_:  This regularization is commonly used to enforce smoothness in imaging applications. The matrix for total variation regularization is defined as follows:
  14 $${{\boldsymbol{\Gamma }}}_{TV}[i,j]=\left\{\begin{array}{ll}1, & \mathrm{if}\ i=j\\ -1, & \mathrm{if}\ i-1=j\\ 0, & \mathrm{otherwise}.\end{array}\right.,\ \mathrm{for}\ 1\le i,j\le n.$$

### Finding the optimal *λ*

The optimal lambda for a given SNR and **Γ** was determined by numerically minimizing the mean squared error of a the 6144 test images using the Broyden-Flecher-Goldfarb-Shanno^26^ algorithm.

### SVD algorithm

In order to achieve the optimal filter weights for the SVD algorithm (described in Section 3.2), we numerically minimized the loss function $mean(| {log}_{10}(\frac{\widehat{\sigma }}{\sigma })| )$, where *σ* denotes the power of the noise, and $\widehat{\sigma }$ is its estimate. We trained the Broyden-Flecher-Goldfarb-Shanno algorithm across the 18432 training images with logarithmically distributed *σ* such that the SNR varies from 0 dB to 60 dB. After doing the training algorithm, we found an optimal filter of size 5 and weights equal to [−3.365, 6.881, −0.506, −3.417, 1.402]. As depicted in Fig. 3c, exploiting the optimal filter for SVD improves the performance significantly compare to the symmetric filter $\left(\left[-\frac{1}{3},\frac{2}{3},-\frac{1}{3}\right]\right)$.

### CNN algorithm training

We trained a Convolutional neural network with the following architecture:

- Layer 1: 1D Convolution; 30 filters of size 51, activation: sigmoid, padding: same size. 1D Max Pooling; Pool size = 4
- Layer 2: 1D Convolution; 5 filters of size 15, activation: sigmoid, padding: same size
- Layer 3: Fully Connected layer; 25 neurons, activation: sigmoid
- Layer 4: Fully Connected layer; 15 neurons, activation: sigmoid. Fully Connected neuron; 1 neurons, activation: exponential

The CNN was trained using the Adam optimizer to minimize $mean(| {log}_{10}(\frac{\widehat{\sigma }}{\sigma })| )$ with logarithmically distributed *σ* across the 18432 training images.

## Supplementary information

Supplementary Information.
