# Supplementary material for: A Silicon Photonics Computational Lensless Active-Flat-Optics Imaging System
Source: Sci Rep. 2020 Feb 3;10:1689. doi: 10.1038/s41598-020-58027-1 (PMC6997425; doi:10.1038/s41598-020-58027-1)
Supplement: Supplementary file 1 — Supplementary Information. [file 41598_2020_58027_MOESM1_ESM.pdf]

Supplemental Information: A Silicon Photonics Computational Lensless Active-Flat-Optics Imaging System

Alexander White*[1], Parham Khial[1], Fariborz Salehi[1],
Babak Hassibi[1], Ali Hajimiri[1]

Grating Coupler Information

The 20 grating couplers we use each have 50 uniformly spaced gratings. Each grating consists of a 300um long unetched 220nm silicon section and a 160um etched silicon section of variable length. The length of the etched section of each grating coupler is modulated linearly from 260nm to 440nm. The gratings are 3um wide and are mode matched to a 500nm wide by 220nm tall single mode transverse-electric waveguide with a 3um long linear taper. Both the waveguides and gratings are oxide clad and capped. Using finite-difference-time-domain (FDTD) simulation, we can estimate the peak efficiency of each coupler, Supplemental Figure 1.

The spectral bandwidth of the grating couplers is around 350nm, and far exceeds the 50nm bandwidth of the super-luminescent diode used for measurements. As the grating couplers are uniform, this bandwidth is set primarily by the reception angle: as the peak reception angle tends towards ±90deg, the grating couplers' efficiency drops. However, the couplers are still receptive, albeit with a much reduced efficiency, to much higher and lower wavelengths as the interference condition given in equation 1 can still be met.

Mapping SNR to λ_{opt}

To reconstruct an image based on its predicted SNR, we must map this SNR to a regularization strength λ. While the optimal mappings are close to linear, a linear approximation is not exact, Supplemental Figures 2 and 3. Thus, for optimal reconstruction, we can use more simulated mappings between SNR and λ. These can be generated by numeric optimization of λ across a set of noisy data.

As regularizers perform differently across data types, we generate two sets of mappings. One set maps SNR to λ in natural images, optimized over the 18432 images used in simulations (Supplemental Figure 2a, top). The other set maps SNR to λ in sharp images, like the barcodes physically measured.

These mappings were optimized over 15000 randomly generated barcode images (Supplemental Figure 2a, bottom).

As each $\mathbf{\Gamma}$ matrix will perform differently on a given dataset, a separate mapping is required for each one. While we do need multiple maps, using SNR as a mediator between image analysis and regularization strength allows for a single SNR prediction algorithm for all $\mathbf{\Gamma}$ matrices.

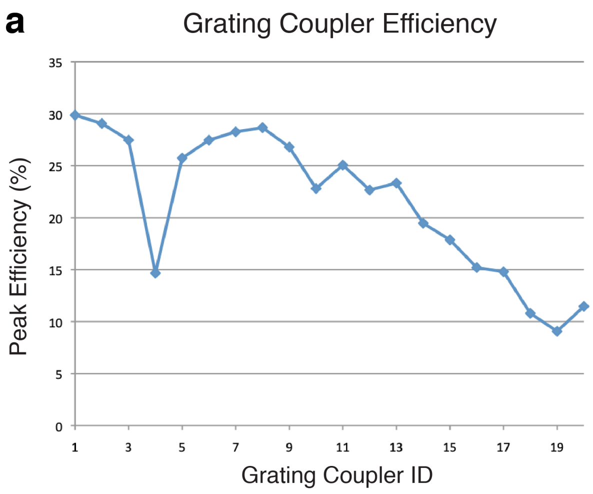

Supplemental Figure 1: a) Peak efficiency of grating couplers. Efficiency drops for the coupler with near perpendicular optimal incidence angle and drops for steeper angles.

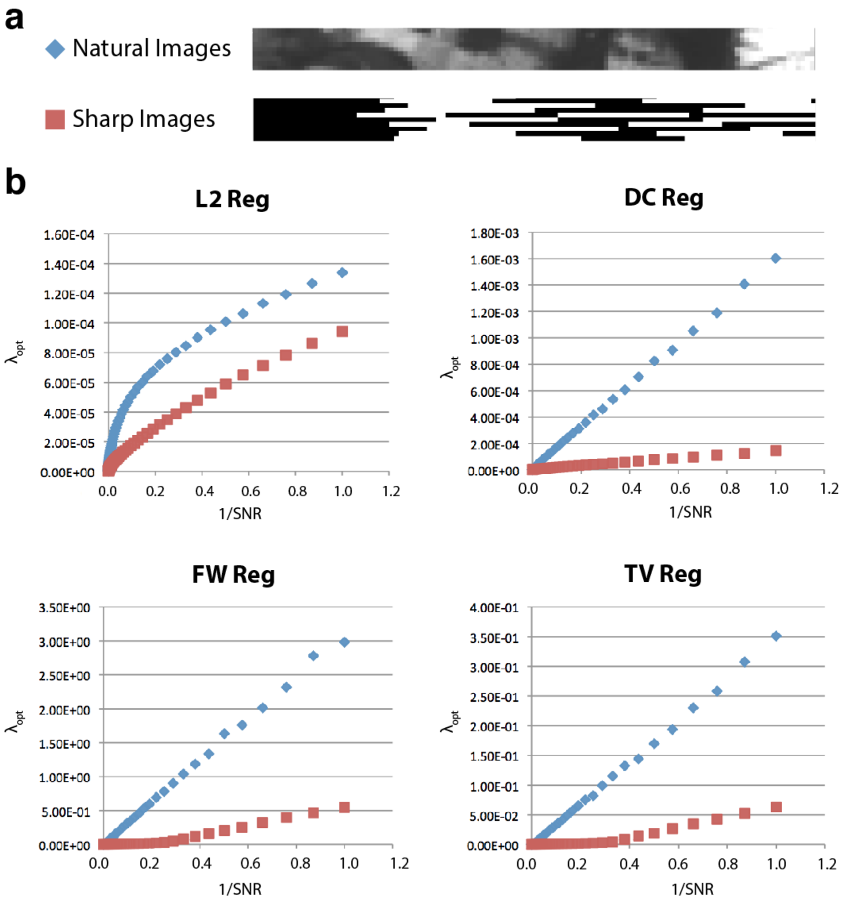

Supplemental Figure 2: a) Examples of natural images and randomly generated sharp images used in 1/SNR to λ_{opt} mapping. b) Plots of the optimal lambda across 1/SNR from 10^{-6} to 1. This mapping is shown for each of the four $\mathbf{\Gamma}$ matrices and for natural and sharp images.

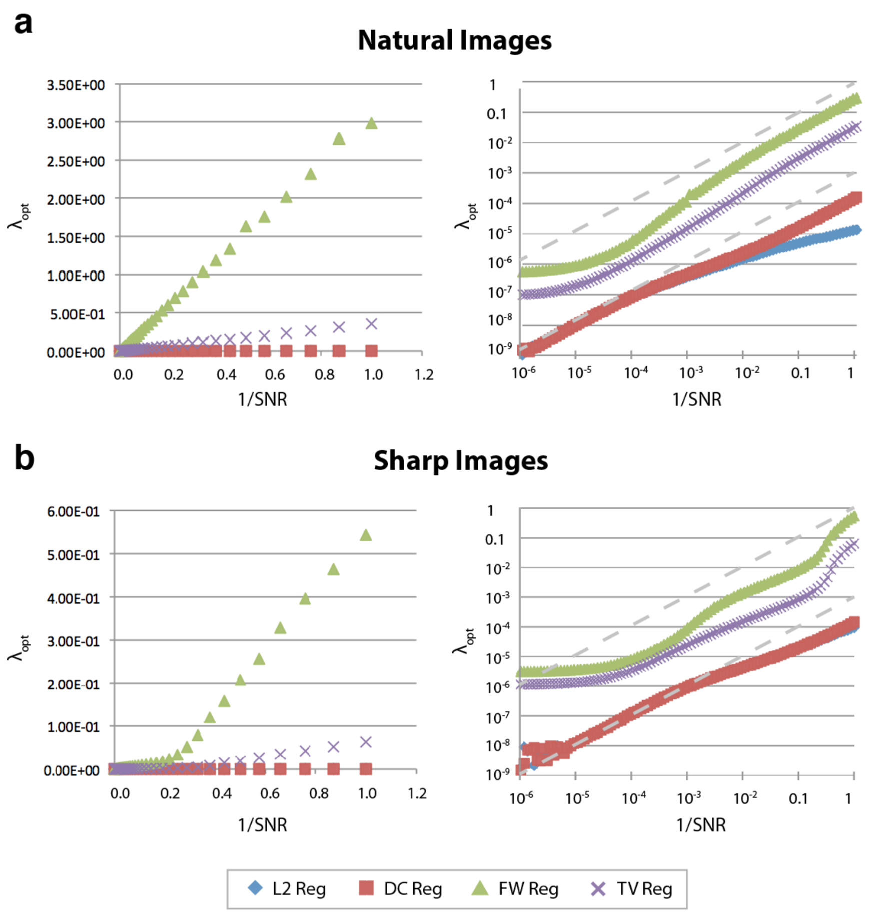

Supplemental Figure 3: a) Linear and log-log plots of 1/SNR to λ_{opt} for natural images using all four $\mathbf{\Gamma}$ matrices. Dashed lines in log-log plot indicate linear slope. b) Linear and log-log plots of 1/SNR to λ_{opt} for sharp images using all four $\mathbf{\Gamma}$ matrices. Dashed lines in log-log plot indicate linear slope.
